# 

**Published:** 1877-10

**Authors:** 


### EDITORIAL.

SPRINGFIELD, MASS., Sept. 18, 1877.

The Fourteenth Annual Meeting of the Connecticut Valley Dental Society, will be held at Haynes' house, Springfield, Massachusetts, on Tuesday and Wednesday, October 23d and 24th, 1877, commencing at 10 1-2 o'clock, A.M., on Tuesday.

C. T. STOCKWELL, *Secretary.*

### TEXT BOOKS.

We have just received of Lindsay & Blakiston, "The Medical Intelligencer" containing comments, editorial notices, and contents of new and important works, recently published with a list of text books, and other publications issued by their house.

Many of them are now sold at greatly reduced prices, in view of the present monetary condition of the country. Many of the most valuable works of Medical and Dental literature are published by this house.

A complete list can be obtained, by simply sending name and address to them.

A MONTHLY MAGAZINE DEVOTED TO THE INTERESTS OF

**THE DENTAL PROFESSION.**

*TERMS REDUCED TO $2.50 PER YEAR. SINGLE COPIES 25C.*

EDITED BY PROF. J. TAFT, D.D.S.

AND

Published by SPENCER, CROCKER & CO., Ohio Dental Depot.

The volume commences in January and closes in December.

The REGISTER being issued monthly is always fresh, therefore Indispensable to the practitioner who desires to keep posted up with the new inventions and improvements which are daily developed. Neither expense nor effort will be spared to give value and variety to its contents. Articles will be illustrated with well executed engravings whenever they will add to the interest or assist to explanation.

Its extensive circulation and frequency of issue make it one of the BEST DENTAL ADVERTISING MEDIUMS in the United States.

All subscriptions must begin with January or July.

Local or special notices, five lines or less, will be inserted for $3 each insertion; over five lines, 50 cts. per line.

All advertising bills payable in advance, or at furthest, after the issue of the first number containing the advertisement. All transient advertisements to be paid for in advance.

☞CONTRIBUTIONS SOLICITED.

Exclusively Editorial Communications should be addressed to Editor.

The latest number of the REGISTER may be ordered with or without other goods at any time, and all letters should be addressed to

***SPENCER, CROCKER & CO., Ohio Dental Depot, Cincinnati, O.***

